# Quantifying bacterial efflux within subcellular domains of *Pseudomonas aeruginosa*

**DOI:** 10.1128/aem.01447-24

**Published:** 2024-10-30

**Authors:** Yujie Li, Michael J. Wilhelm, Tong Wu, Xiao-Hua Hu, Oscar N. Ruiz, Hai-Lung Dai

**Affiliations:** 1 Institute for Membranes and Interfaces, Temple University, Philadelphia, Pennsylvania, USA; 2 Department of Chemistry, Temple University, Philadelphia, Pennsylvania, USA; 3 Biomaterials Branch, Materials & Manufacturing Directorate, Air Force Research Laboratory, Wright-Patterson AFB, Ohio, USA; Danmarks Tekniske Universitet The Novo Nordisk Foundation Center for Biosustainability, Kgs. Lyngby, Denmark

**Keywords:** efflux pump, Gram-negative bacteria, second harmonic generation, biodeterioration, biodegradation

## Abstract

Molecular efflux is a mechanism through which bacteria actively expel undesirable substances. This is a crucial line of defense against toxic chemicals in harsh environments. Understanding how efflux works is critical for designing antimicrobial strategies. Though much is already known about efflux proteins, important details about the mechanisms of efflux (e.g., importance of specific subcellular domains and ejection rates) have yet to be experimentally quantified. Herein, we use the nonlinear optical technique, second harmonic light scattering, to simultaneously measure the efflux rates from the periplasm and cytosol of a Gram-negative bacterium. The influence of efflux on the uptake kinetics of a mild antimicrobial, malachite green (MG), by *Pseudomonas aeruginosa* was quantified. It is observed that efflux primarily occurs from the periplasm and is two orders of magnitude faster than from the cytosol. Efflux pumps activate to maintain MG concentrations in the periplasm below 1 µM, while efflux from the cytosol maintains MG concentration below 0.1 µM. Efflux pumps are shown to saturate when exogenous MG concentrations are greater than 25 µM, while the cytosol efflux function saturates at >15 µM. Finally, efflux pumps can simultaneously eject different compounds, as proven by experiments with both MG and hexane, a known effluxable compound.

**IMPORTANCE:**

Molecular efflux pumps are a crucial defense mechanism that protects bacteria from an otherwise unchecked influx of toxic molecules present in the extracellular environment. The efflux functions constitute a significant hindrance to antimicrobial efficacy. While much is now known regarding the structure of these channels, knowledge of the influence of efflux in individual subcellular domains and the associated ejection rates is still lacking. Using the nonlinear optical technique, second-harmonic light scattering, we have measured the threshold concentrations for pump activation, saturation concentrations, and efflux rates from both the periplasm and cytosol in living Gram-negative bacteria. The quantified efflux data in the different subcellular compartments not only provide a clear mechanistic understanding but also are critical for developing antimicrobial strategies.

## INTRODUCTION

Bacteria, as one of the most adaptable and omnipresent forms of life on Earth, have demonstrated an exceptional ability to adapt to challenging environments, with extreme physical parameters such as temperature, pH, and salinity. Bacteria generally prefer environments that offer moderate temperatures, neutral pH levels, and balanced salinity. Nonetheless, a growing number of bacteria have been discovered to thrive in environments that are considerably harsher (1) due to the presence of hydrocarbons, heavy metals, various antibiotics, and a gamut of recalcitrant toxic compounds.

One example of bacterial growth in harsh environments is the biofilm formation in fuel, which is of significant concern to the transportation industry. This phenomenon can lead to detrimental changes in the physicochemical properties and quality of fuel and cause costly deterioration of the fuel system (2–13). Fuel biodeterioration is a big concern for aircraft safety and operation. To effectively mitigate bacterial proliferation in jet fuel and other hydrocarbon fuels, the primary step is to gain an understanding of how these microorganisms adapt to the fuel-containing environment. While both Gram-positive and Gram-negative bacteria have been isolated from fuel-containing environments, Gram-negative bacteria are more prevalent in biodeteriorated jet fuel (7, 14–20). For example, *Pseudomonas aeruginosa* (*P. aeruginosa*) ATCC 33988, *Pseudomonas putida* (*P. putida*), and *Marinobacter hydrocarbonoclasticus* NI9 have been shown to flourish in jet fuel and marine diesel (20).

It has been revealed that bacterial resistance to the extreme hydrocarbon-rich environment is largely achieved by regulating the intracellular concentration of hydrocarbons (21–23), which is attributed to the presence of efflux pumps. Many efflux pump systems have been characterized in bacteria with the resistance–nodulation–division (RND) efflux pump mechanism commonly found in Gram-negative bacteria (24–27). The tripartite RND superfamily efflux pump systems are proton antiporters that undergo the extrusion of toxic compounds from the cell cytoplasm and periplasm to the external environment in order to regulate homeostasis and maintain manageable levels of toxicant in the cell (28–31). The RND efflux pumps are composed of three protein components (32–39): an inner membrane pump that is located on the cytoplasmic membrane, an outer membrane factor (OMF) that serves as the exit for the chemicals, and membrane fusion proteins that are anchored in the inner membrane to connect the OMF and the inner membrane pump. Many of these pumps are nonspecific, effectively expelling a broad spectrum of hydrocarbons and organic solvents (20, 31, 40), antimicrobials and antibiotics (28, 32, 34, 41–44), and dyes and toxins (38, 39, 45). Consequently, the efflux pumps play a significant role in the bacterial intrinsic resistance toward harmful molecular species from the environment. The MexAB-OprM efflux pump of *P. aeruginosa* is shown to provide resistance to a wide range of antibiotics including tetracycline, chloramphenicol, β-lactams, and fluoroquinolones (46). It is postulated that the RND efflux pumps evolved in bacteria as resistance mechanisms to cope with endogenously produced antibiotics and toxic metabolites (30).

Numerous studies have been conducted to understand the functions of bacterial efflux pumps. For instance, the effect of bacterial efflux activity is revealed by biological susceptibility assays comparing minimum inhibitory concentration (MIC) values between wild-type strains and efflux pump mutant strains (i.e., strains lacking efflux pumps) (22, 23, 45, 47). Typically, wild-type strains, which possess more robust efflux activity, exhibit higher MIC values compared to mutant strains with compromised efflux capabilities. This approach has been used to measure antibiotic resistance across various antimicrobials (48). However, the absence of kinetic information regarding the transport of these antibiotics limits the interpretation of these results.

Fluorescence-based techniques have been utilized to observe the intracellular accumulation of molecules in real time and subsequently assess the performance of the efflux pump (24, 49–55). Nevertheless, these methods face a significant challenge: At high concentrations, the probe molecules used for fluorescence can experience quenching within the cell membrane. This phenomenon hinders the accuracy of correlating fluorescent intensity with the actual concentration of the probe molecules. Moreover, the complex structure of the Gram-negative bacterial cell envelope, which includes an outer membrane (OM), a peptidoglycan mesh (PM), and a cytoplasmic membrane (CM), presents another challenge. The fluorescence-based methods lack the necessary resolution to distinguish between these multiple membranes, making it difficult to precisely track the movement of molecules across the intricate layers of the Gram-negative cell envelope.

Understanding the kinetic behavior of the RND efflux pump in Gram-negative bacteria is crucial for elucidating how it contributes to their defense mechanisms against harsh environmental conditions. Structural biology studies of the RND efflux pump protein (29, 56, 57) *in Escherichia coli* (*E. coli*), a homolog of MexB in *P. aeruginosa*, suggest that the pump protein of the efflux pump captures unwanted molecules from both the outer and inner leaflets of the cytoplasmic membrane so that it can lower the intracellular concentration in both the cytosol and periplasmic regions. Nevertheless, experimentally characterizing the kinetic behavior of RND efflux pumps is challenging. To date, there has only been a study on periplasmic efflux. Specifically, Nagano and Nikaido (26) reported *in vivo* monitoring of the kinetics of the RND efflux pump in removing various β-lactams from the periplasm. The efflux rate from the periplasm was inferred from the observed hydrolysis rate of β-lactams by periplasmic β-lactamase and the calculated influx rate into the periplasm. However, molecular efflux from the cytosol has not yet been observed. To quantitatively ascertain the effect of an efflux pump, there remains a critical need for an effective experimental platform that can track molecules transporting across the bacterial cell membranes individually in real time.

In this study, we show that the nonlinear optical technique, second harmonic (SH) light scattering (SHS), can be used to observe in real time the translocation of molecules across individual membranes in living bacteria. More importantly, these experiments can be done while efflux pumps are active, such that it is possible to separately quantify the efflux rates from both the periplasm and the cytosol.

SHS is based on the nonlinear optical phenomenon, second harmonic generation (SHG), in which a portion of the fundamental light (when interacting with noncentrosymmetric media) is converted to light with twice the original frequency. The application of SHS to measure molecular adsorption and transport at a lipid bilayer membrane is as follows (58): Molecules without center of inversion symmetry can facilitate SHG and are referred to as SH-active molecules. The SH optical fields generated by an ensemble of such molecules in random orientation, such as in a liquid solution, cancel with one another; therefore, no coherent SH light is detected. When SH-active molecules adsorb onto the surface of a membrane, they align with one another via the same molecule–membrane interactions. This alignment results in constructive interference among the SH fields and gives rise to a detectable SH signal. The intensity of the signal is quadratically proportional to the number density of molecules adsorbed on the surface. As molecules transport across the membrane, they can adsorb onto the inner leaflet of the membrane. While aligned with each other, these molecules have opposite orientations with respect to the molecules on the outer leaflet. The SH fields generated by the molecules adsorbed on the inner leaflet cancel the fields from the outer leaflet, causing a decrease in the detected SH signal. In this process, the rise and the decay in the time-resolved SH signal can be attributed to the adsorption of molecules on the outer leaflet of the membrane followed by the transport and adsorption of the molecules to the inner leaflet. This interpretation of the time-dependent SHS kinetic response has been extensively validated in numerous prior studies of liposomes (59–67) and living cells (both eucaryotic and bacterial) (68–79) and quantitatively corroborated in complementary studies using time-resolved brightfield transmission microscopy (70, 80).

SHS has several advantages over other methodologies: It typically uses infrared light as an excitation source, minimizing damage to the biological sample. SHG is a coherent nonlinear optical phenomenon, differing from two-photon excited fluorescence, which involves photoabsorption, and is free from photobleaching. Initially demonstrated by Eisenthal and coworkers for observing transport across liposome membranes (59, 60, 62, 81–83), SHS has since been applied to the study of molecular adsorption and transport within multi-membrane systems (68, 69, 71, 73, 76, 77, 79, 80, 84–86) and is now employed by laboratories worldwide to monitor molecular interactions with biological membranes (74, 75, 87–89).

Herein, we apply SHS to study the effect of efflux pumps on the uptake of the hydrophobic ion, malachite green (MG), across the membranes in *P. aeruginosa* ATCC 39327, a wild-type strain of *P. aeruginosa*. MG is an SH-active molecule and belongs to the family of quaternary ammonium cations, which have known antibiotic effects (90, 91) and typically serve as the active ingredient in commercial disinfectants (e.g., Lysol). We first examine the influence of efflux pumps on the molecular uptake of increasing concentrations of MG in *P. aeruginosa*, as this enables the quantitative determination of the efflux removal of MG from both the periplasm and the cytosol. Next, MG uptake is measured in the presence of an excessive concentration of an (SH-inactive) effluxable compound (i.e., hexane), in order to overwhelm the efflux pumps and further illustrate the influence of the efflux pump.

## RESULTS

### Time-resolved SHS from MG interacting with *P. aeruginosa*

**Fig 1:**
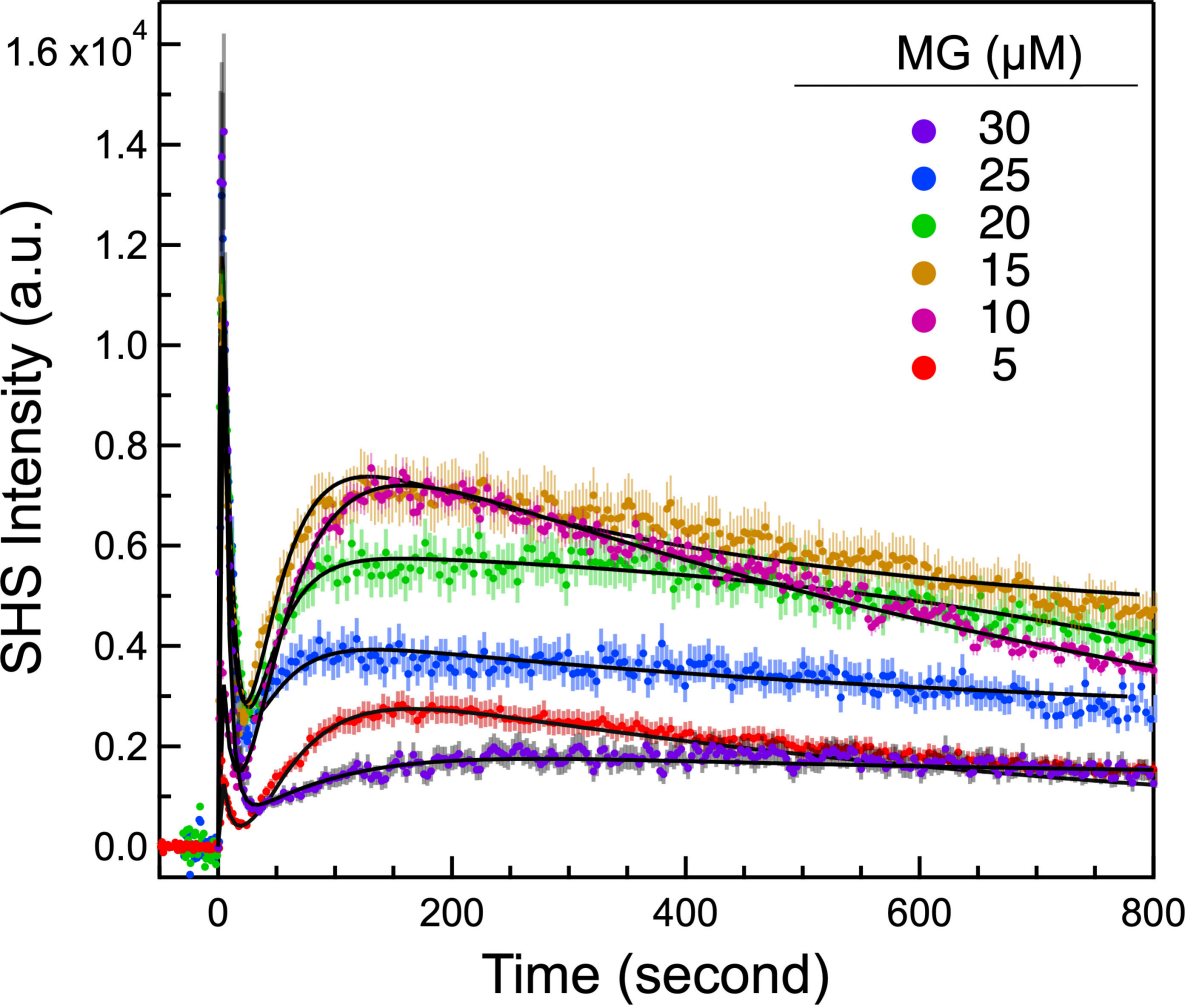
Time–resolved SHS traces observed following the addition of *P. aeruginosa* (at *t* = 0 s) into solutions containing MG, for specified concentrations. Solid black lines show the optimal fitting results using the kinetic model including efflux.

We first examine the MG concentration-dependent adsorption and transport in *P. aeruginosa*. Figure 1 shows the representative time-resolved SH signals recorded from samples containing MG at different concentrations after adding the bacterial samples. The sharp rise of the first peak is attributed to MG’s rapid adsorption onto the OM’s outer surface. The fast decay is the consequence of rapid MG transport across the porin channels of the OM and adsorption onto the inner surface of the OM. MG molecules adsorbed to the opposite surfaces of a membrane would have opposite orientations, and the SH light generated from the opposing surfaces cancels with each other, causing the decay of the first SH peak. In contrast, the slow rise of the second broad peak stems from the diffusion of MG through the PM, followed by slow adsorption onto the outer surface of the CM. Finally, the slow decay of the second peak is the result of passive diffusion of MG across the CM, a process that is typically more than two orders of magnitude slower than MG transport across the porin channels (68, 77, 92, 93).

The SHS traces in Fig. 1 show that as the MG concentration increases, the magnitude of the first peak increases. In contrast, the CM peak (i.e., the second transport peak) increases only up to MG concentrations of 10 µM and begins to decrease after 15 µM. The decay rate of the CM peak is observed to slow down (become less steep) as the MG concentration increases, indicating that the CM transport rate constant decreases with increasing concentrations of MG. This phenomenon is different from the previously reported CM transportation behavior in *E. coli* (75). A plausible explanation for the observed MG concentration-dependent decay rate in the CM is the active involvement of the efflux pumps in this process: the efflux pumps are activated and expel MG molecules when the concentration reaches a threshold value, reducing the apparent molecular transport rate.

### Determination of efflux rates from individual compartments within *P. aeruginosa*

A kinetic model that incorporates adsorption and transport across the OM and CM as well as transport through the PM was used to analyze the measured SHS traces. This model has been shown effective in extracting the adsorption densities and transport rates at all membranes of a bacterium (68, 71, 76, 77, 80) lacking efflux pumps. Nevertheless, the original kinetic model was insufficient to accurately model the SHS kinetic traces in Fig. 1, as the resulting fits gave nonphysical results (e.g., showing a decrease in the membrane transport rate constant with increasing concentration). In order to explain the observed concentration-dependent SHS traces in Fig. 1, the kinetic model needed to be updated to include the effect of efflux pumps on molecular concentrations in the periplasm and cytosol.

**Fig 2:**
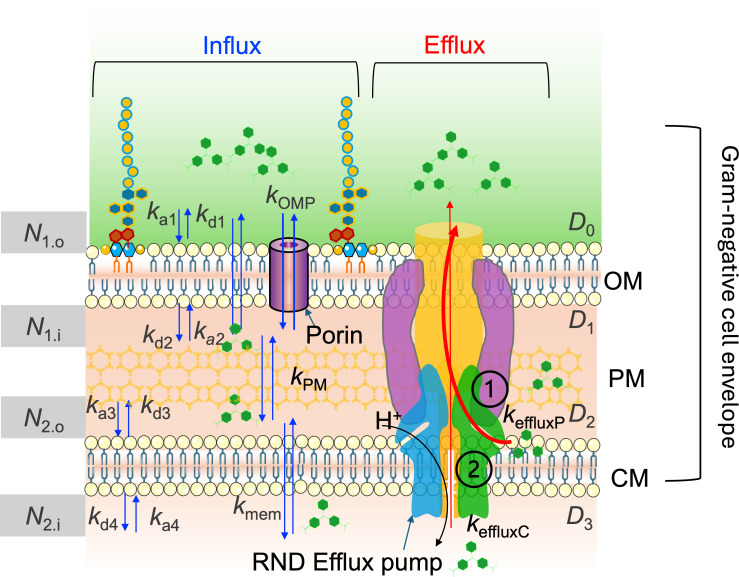
Schematic representation of the kinetic model describing molecular uptake in *P. aeruginosa*, including molecular influx and efflux. In the model, *N*_1_*_.o_, N*_1_*_.i_, N*_2_*_.o_,* and *N*_2_*_.i_* represent the maximum number density of MG adsorbed on the outer leaflet of the OM, inner leaflet of the OM, outer leaflet of the CM, and inner leaflet of the CM, respectively. *D*_0_*, D*_1_*, D*_2_*,* and *D*_3_ represent the concentration of MG in each compartment depicted with different shades of yellow. *k_a_*_1_*, k_d_*_1_*; k_a_*_2_*, k_d_*_2_*; k_a_*_3_*, k_d_*_3_*;* and *k_a_*_4_*, k_d_*_4_ represent the adsorption and desorption rate constant on the outer leaflet of OM, inner leaflet of the OM, outer leaflet of the CM, and inner leaflet of the CM, respectively. *k*_OMP_*, k*_PM_*,* and *k*_mem_ represent the rate constant for crossing OM porin channel, PM, and CM membrane diffusion, respectively. *k*_effluxP_ represents the efflux rate constant of sending molecules back to the extracellular space from the periplasmic region, and *k*_effluxC_ represents the efflux rate constant of sending molecules back to the extracellular space from the cytosol region. Two potential efflux behaviors are shown in two pathways; pathways ① and ② represent pumping molecules to the extracellular space, either from the periplasmic region (#1) or from the cytosol (#2), respectively.

Figure 2 depicts the comprehensive kinetic model, which now accounts for efflux through two specified pathways: pathway 1 through the periplasm and pathway 2 through the cytosol. The two pathways depict the efflux mechanism transporting molecules from these two compartments back into the extracellular space. The presence of the efflux function introduces competition between the efflux activity and the adsorption processes at the membranes of the corresponding intracellular regions. In addition to the equations in the original kinetic model described in the supporting information (S4–S11), the updated model includes the efflux processes of the two pathways delineated through equations (1) and (2) and 2:


(1) $$D_{2}\overset{k_{effluxP}}{\to }\;D_{0},$$

(2) $$D_{3}\overset{k_{effluxC}}{\to }\;D_{0},$$

where *k*_effluxP_ and *k*_effluxC_ represent the rate constants for efflux from the periplasm and cytosol, respectively, and *D_0_* is the extracellular MG concentration. Compared with the previous version of the kinetic model, there are two more parameters in addition to the rate constants in the updated model: *D*_2_ is the MG concentration in the region bounded by the PM and the CM, and *D*_3_ is the MG concentration in the cytosol.

The SHS traces in Fig. 1 were fit to the updated kinetic model in a global fit analysis by assuming that the maximum number density on each leaflet (i.e., $N_{1,o}^{Max}$*,* $N_{1,i}^{Max}$*,* $N_{2,o}^{Max}$*,* and $N_{2,i}^{Max}$), the adsorption free energy on each leaflet (i.e., *∆G*_1_*_,o_*, *∆G*_1_*_,i_,* and *∆G*_2_*_,o_*), and the transport rate constants across the OM porin channels, the PM, and the CM (i.e., *k*_OMP_, *k*_PM_, and *k*_mem_) remain the same across all concentrations. Only *k*_effluxP_ and *k*_effluxC_ are allowed to change. The deduced rate constants, including *k*_effluxP_ and *k*_effluxC_, and other parameters, along with their respective standard deviations, are listed in Table 1. Notably, *k*_effluxP_ exhibits an increasing trend with higher concentrations but plateaus at concentrations above 25 µM. Similarly, *k*_effluxC_ follows an increasing trend with MG concentrations but appears to reach a maximum at concentrations larger than 15 µM.

**TABLE 1:** Transport rate constants deduced as fitting parameters in the complete kinetic model (including efflux) for the data presented in Fig. 1^*a*^

[MG^+^] (µM)	5	10	15	20	25	30
*k*_OMP_ (×10^−2^ s^–1^)*^b^*^,*d*^	7.9 ± 0.4	7.9 ± 0.4	7.9 ± 0.4	7.9 ± 0.4	7.9 ± 0.4	7.9 ± 0.4
*k*_mem_ (×10^−3^ s^−1^)^*b*^^,*d*^	1.1 ± 0.5	1.10 ± 0.5	1.1 ± 0.5	1.1 ± 0.5	1.10 ± 0.5	1.1 ± 0.5
*k*_PM_ (×10^−2^ s^−1^)*^b^*^,*d*^	2.5 ± 0.3	2.5 ± 0.3	2.5 ± 0.3	2.5 ± 0.3	2.5 ± 0.3	2.5 ± 0.3
*k*_effluxP_ (s^−1^)^*c*^	(3.9 ± 2.7) × 10^−2^	(4.7 ± 1.4) × 10^−2^	0.20 ± 0.1	0.5 ± 0.2	1.3 ± 0.5	1.2 ± 0.5
*k*_effluxC_ (s^−1^)^*c*^	(2.3 ± 1.1) × 10^−6^	(1.0 ± 0.2) × 10^−3^	(4.7 ± 1.0) × 10^−3^	(4.8 ± 1.1) × 10^−3^	(5.0 ± 1.2) × 10^−3^	(5.0 ± 1.1) × 10^−3^

^*a*^ Error bars correspond to the standard deviation obtained from the fitting.

^*b*^ *k*_OMP_*, k*_PM_*,* and *k*_mem_*,* as depicted in Fig. 2, represent the rate constant for crossing OM porin channel, the peptidoglycan mesh, and membrane diffusion, respectively**.**

^*c*^ *k*_effluxP _represents the rate constant for pumping molecules back to the extracellular space from the periplasmic region. *k*_effluxC_ represents the rate constant for pumping molecules back to the extracellular space from the cytosol region.

^*d*^ The fit analysis is conducted by means of global fit where all the parameters, along with their respective standard deviations, are consistent across the concentrations, except for the parameters *k*_effluxP_ and *k*_effluxC_.

### Efflux of hexane

It has previously been shown that linear alkanes can be ejected by the MexAB efflux pump in strains of *P. aeruginosa* (21, 23, 94, 95). Consequently, strains of *P. aeruginosa* with efflux function exhibit lower susceptibility to linear alkanes. For example, Gunasekera et al. found that treating *P. aeruginosa* (ATCC 33988) cells (growing in Jet A fuel) with the efflux pump inhibitor (EPI), PAβN, resulted in growth inhibition. Conversely, untreated strains of *P. aeruginosa* continued to thrive in the fuel-rich environment (21, 22). Given that efflux pumps are typically nonspecific (i.e., they pump out a wide variety of undesirable compounds), it is anticipated that alkane molecules and MG will be removed by the same efflux pumps. As a result, linear alkane chains may compete against MG to be effluxed and, hence, influence the apparent transport rate of MG crossing the CM.

The short-chain alkane, n-hexane, was selected to test this hypothesis because it has a desirable solubility in water (110 µM), and it is not likely degraded by *P. aeruginosa* ATCC 39327. Previous studies have shown that *P. aeruginosa* have a preference for medium-chain alkanes between n-C10 and n-C18 and cannot degrade shorter alkanes such as n-C6 to n-C8 (20, 22, 96). After resuspending the cells in 1× phosphate buffered saline (PBS) containing 110 µM hexane, the cells and hexane are in a state of coexistence. Subsequently, the SHS experiments were repeated to investigate MG uptake in the cells at both low (5 and 10 µM) and high (25 and 30 µM) MG concentrations. Unlike MG, hexane is neutral and does not compete with MG for surface adsorption sites. Furthermore, n-hexane does not generate a measurable SH response. Most importantly, hexane has been identified as a substrate of the efflux pump. Prior studies have shown that wild-type *P. aeruginosa* can thrive in environments with n-hexane concentrations exceeding 110 µM (94, 97). However, mutants lacking efflux pumps are unable to grow under similar hexane conditions (94). This suggests that the relatively low susceptibility of the wild-type bacteria to hexane can be partially explained by the active role of the MexAB efflux pump and potentially other RND efflux pumps in expelling hexane from the cell interior.

The experiments reported here use high concentrations of hexane with the specific intent of saturating the efflux pump. Given the significantly higher concentration of hexane, the efflux pump should be operational to maintain a low concentration of hexane inside the cell, thereby limiting its availability to transport other molecules (such as MG). In this scenario, the two different types of exogenous molecules create competition for efflux activity.

**Fig 3:**
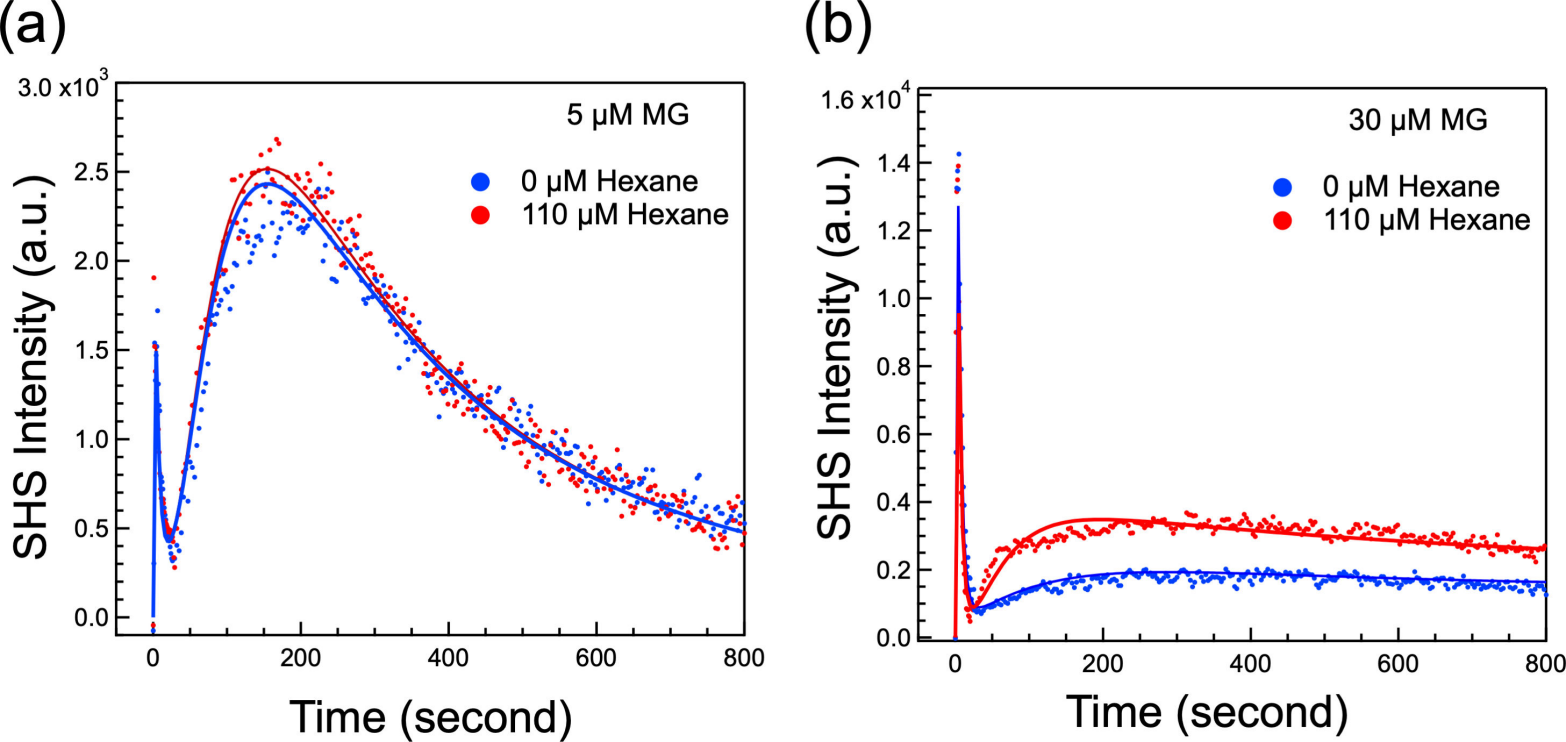
Measured time-resolved SHS traces for (a) 5 µM MG (1.6 µg/mL) and (b) 30 µM MG (9.9 µg/mL). In both panels, red points represent cells pretreated with 110 µM hexane, whereas blue points are from cells untreated with hexane. Solid lines show the optimal fitting of the SHS traces using the updated kinetic model.

**Fig 4:**
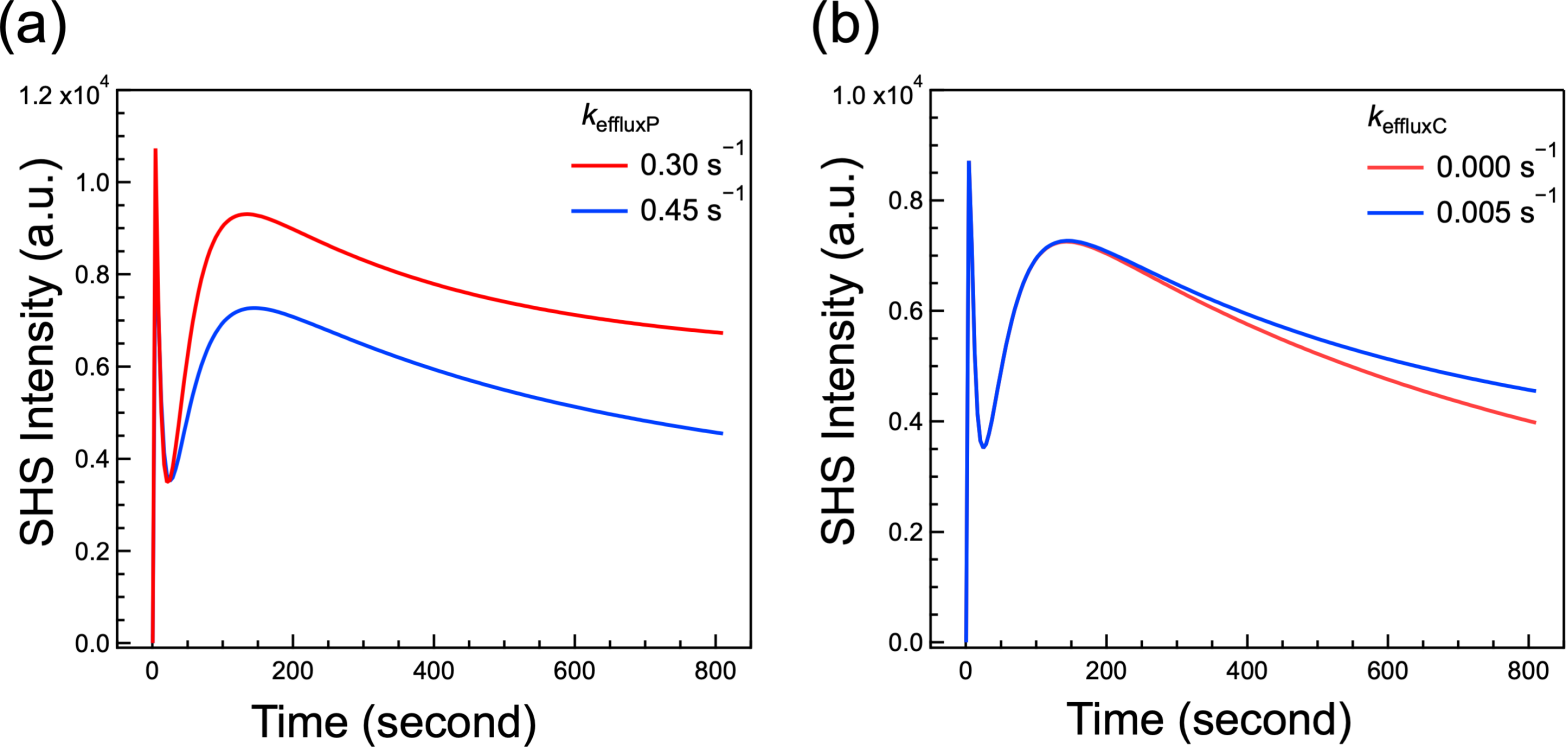
Simulated SHS traces using the updated kinetic model to selectively change the (a) rate constant for periplasmic efflux (*k*_effluxP_) and (b) rate constant for cytosolic efflux rates (*k*_effluxC_). All other parameters are held constant and assigned the values deduced from the fit analysis of the 20 µM sample.

**Fig 5:**
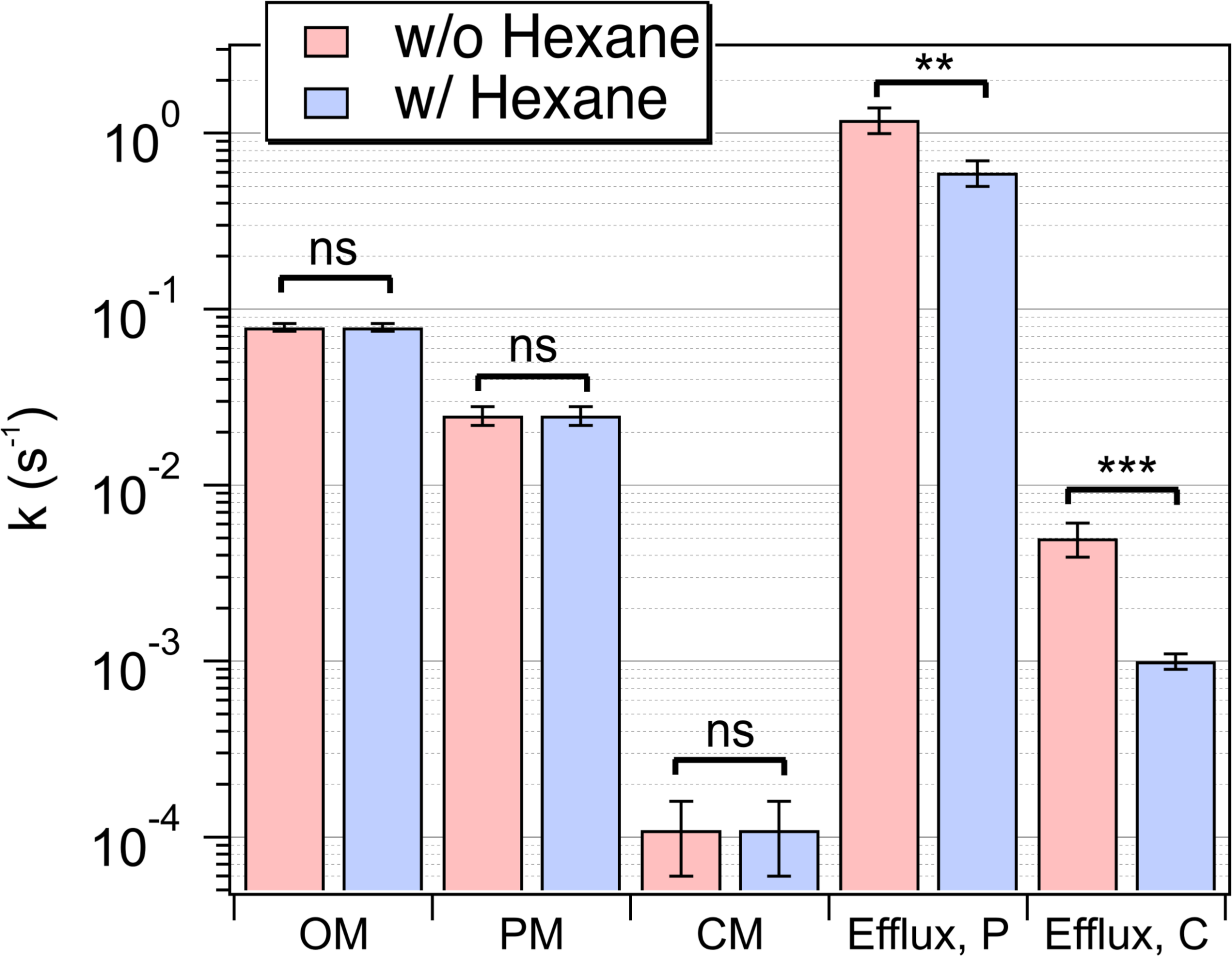
Transport rate constants deduced from fit analysis of the SHS traces (Fig. 3b) for bacterial uptake of MG in the relative presence of hexane. The rate constants correspond to transport across the bacterial OM, PM, CM, and efflux from the periplasm (Efflux P) and cytosol (Efflux C). Error bars represent the standard deviation obtained from the fit analysis (ns: not significant *P* > 0.05, ^*^P ≤ 0.05, ^**^P ≤ 0.01, and ^***^P ≤ 0.001).

In Fig. 3a, the SHS traces indicate that the presence of hexane has virtually no effect on the uptake kinetics of 5 µM MG. The two kinetic responses (i.e., with and without hexane) are effectively superimposable. Conversely, for the uptake kinetics of 30 µM MG (Fig. 3b), we observe a significant change in the CM transport response. Specifically, when hexane is present (red trace), we observe faster adsorption onto the exterior surface of the CM and an increase in the magnitude of the CM transport peak. Observations made with MG concentrations of 10 and 25 µM (Fig. S3) show a similar trend where the 110 µM hexane has no effect on MG transport at 10 µM but a substantial effect at 25 µM. The perturbation of hexane on the transport behavior at high MG concentrations is consistent with the prediction in our simulation shown in Fig. 4a that the reduction of the efflux rate from the periplasm will lead to an increase in the CM peak magnitude. Moreover, the high hexane concentration experiments clearly show the competition of hexane against MG at high concentrations for efflux. This observation further supports the notion that to properly account for the SHS traces, the kinetic model must include efflux pumps. By employing the kinetic model to fit the traces presented in Fig. 3b, we extract parameters related to the molecular transport of MG and quantitatively reveal the effect of a high concentration of n-hexane on efflux functions. The fitting results in Fig. 5 show that *k*_OMP_, *k*_mem_, and *k*_PM_ remain consistent across the conditions as hexane has little impact on MG uptake at low concentrations (5 and 10 µM).

Significantly, when comparing the fitting parameters derived from the uptake kinetics of 30 µM MG with vs without n-hexane, a distinct difference emerges in the rate constants for efflux from both the periplasmic and cytoplasmic regions. Specifically, the presence of high-concentration n-hexane substantially suppresses efflux of MG; *k*_effluxP_ for MG decreases from 1.2 to 0.6 s^−1^ while *k*_effluxC_ decreases from 5.0 × 10^−3^ to 1.0 × 10^−3^ s^−1^. The *k*_effluxP_ derived from data sets with hexane (*M* = 1.2, SD = 0.2) compared with the control group without hexane (*M* = 0.6, SD = 0.1) demonstrated a significant difference, *t*(264) = 2.68, *P* = 0.0078. Similarly, the *k*_effluxC_ derived from data sets with hexane (*M* = 0.005, SD = 0.0011) compared with the control group (*M* = 0.001, SD = 0.0001) also demonstrated a significant difference, *t*(264) = 3.62, *P* = 0.00035.

## DISCUSSION

### Quantifying efflux activities

Prior studies on RND-type efflux pumps (29, 98–100) have shown that pump proteins such as MexB in *P. aeruginosa* and AcrB in *E. coli* facilitate the direct exodus of substrates from the periplasm. Our results suggest that these proteins are also capable of mediating the translocation of substrates from the cytoplasm. Notably, with increasing concentrations of MG, we observe the activation of the efflux pump, leading to the expulsion of MG from both the periplasmic and cytosolic regions. We first discuss the sensitivity in the quantitative assessment of the two efflux pathways characterized by the two rate constants *k*_effluxP_ and *k*_effluxC_ determined from the SHS traces.

The impact of the fitting parameters on the SHS trace can be visualized through model simulation. The full kinetic model including the activity of the efflux pumps from the two different compartments, detailed in the supplemental materials (S4 – S13), consists of 10 processes and 16 parameters. The simulation extends the eight coupled differential equations incorporated in the original model (without efflux), shown in the supplementary information (S14 – S22). We first determine all the parameters, including the efflux rate constants, from fitting the SHS trace acquired from the sample with 20 µM MG. In this fitting, *k*_effluxP_ = 0.45 s^−1^ and *k*_effluxC_ = 0.0005 s^−1^ are determined along with the other 14 parameters. The SHS curve corresponding to this set of parameters is shown in Fig. 4 (blue trace). For comparison, we also simulated scenarios where the efflux pump is less active by setting *k*_effluxP_ = 0.30 s^−1^ or *k*_effluxC_ = 0 s^−1^, respectively, while keeping all other 15 parameters unchanged. As shown in Fig. 4a, increasing efflux through the periplasm (i.e., increasing *k*_effluxP_ from 0.30 to 0.45 s^−1^) leads to a considerable decrease in the magnitude of the peak representing the CM transport. Additionally, our simulation results suggest that efflux from the cytosol will cause the CM transport signal to decay faster, as shown in Fig. 4b. Consequently, the simulations show that the time-resolved SHS traces are sensitive to efflux from both the periplasm and the cytosol.

If efflux is not included in the kinetic model, the analysis yields results that are unphysical. Specifically, we observe that *k*_mem_ decreases as the MG concentration increases when the efflux pump is not included in the kinetic model. For instance, when the external MG concentration is 30 µM, *k*_mem_ decreases by approximately 75% compared to its value for 5 µM MG. Moreover, the adsorption equilibrium constant at the CM also decreases with increasing MG concentration. Both the CM diffusion transport rate constant and the adsorption equilibrium constant should remain constant regardless of MG concentration. These observations highlight the importance of including the functions of efflux in evaluating molecular uptake for bacteria with active efflux pumps.

### Efflux pumps work harder in the periplasm but saturate at higher MG concentrations

**Fig 6:**
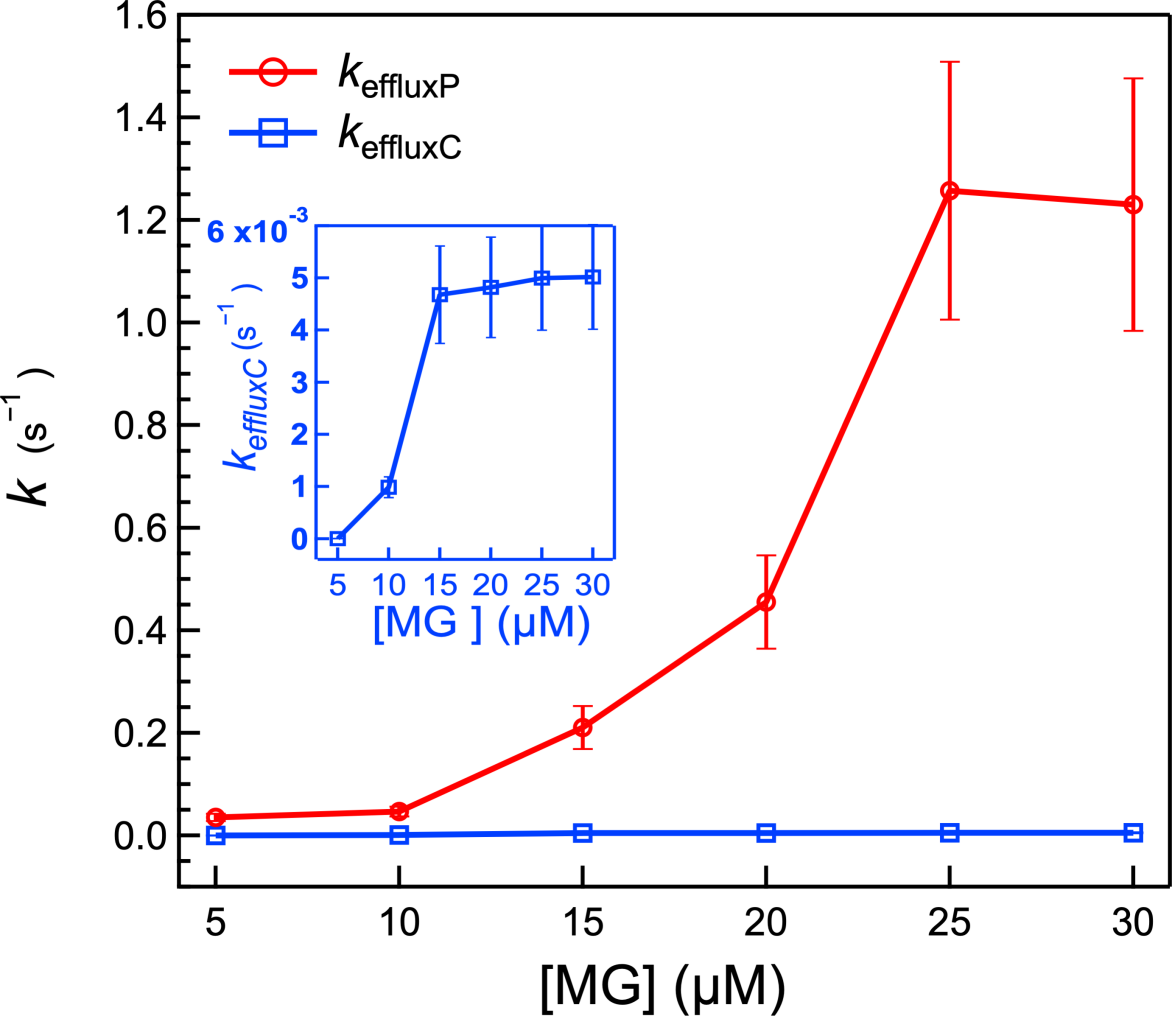
Concentration-dependent behavior of the efflux rates, *k*_effluxC_ and *k*_effluxP_. The inset shows the concentration-dependent behavior of *k*_effluxC_, with a specifically adjusted *y*-axis for clarity. As the concentration of MG varies, *k*_effluxC_ increases from 2.3 × 10^−6^ to 0.5 × 10^−2^ s^−1^, plateauing from 15 to 30 µM, while *k*_effluxP_ rises from 0.04 to 1.3 s^−1^ over the 5–25 µM range, plateauing from 25 to 30 µM. Notably, *k*_effluxP_ surpasses *k*_effluxC_ by two orders of magnitude, indicating the dominant role of efflux from the periplasm. Error bars correspond to the standard deviation of the parameters obtained from the fitting.

The varying magnitude of *k*_effluxP_ and *k*_effluxC_ over the range of exogenous MG concentrations, as shown in Fig. 6, reveals the efflux pump capacities and sensitivities toward MG in the periplasm and cytosol. At low MG concentrations (5 µM), *k*_effluxC_ is notably low at 2.25 × 10^−6^ s^−1^, which is three orders of magnitude smaller than the membrane diffusion rate constant, suggesting efflux insensitivity toward low-concentration MG inside the cytosol. As exogenous MG concentration increases to 10 µM, *k*_effluxC_ increases to 9.88 × 10^−4^ s^−1^ and continues to increase until it plateaus at 4.68 × 10^−3^ s^−1^ for concentrations above 15 µM. Likewise, *k*_effluxP_ increases from 3.35 × 10^−2^ s^−1^ at 5 µM to 1.26 s^−1^ at 25 µM, stabilizing at concentrations above 25 µM. The increase of these rate constants with increasing MG concentration indicates that the efflux pump responds to the environment (i.e., the cell may change the transporter conformation (98) to increase its pump capacity when more undesirable molecules diffuse into the cytosol). However, there is a limit for the efflux pump capacity: The plateauing of *k*_effluxC_ and *k*_effluxP_ at concentrations above 15 and 25 µM, respectively, implies that the channel size for the transporter conformation reaches a maximum, or up-regulation of the number of transporters has been reached. It is noted that *k*_effluxP_ is about two orders larger than *k*_effluxC_, implying that the elimination of MG predominantly occurs via efflux from the periplasm. Although the cytosolic efflux pathway is lower in magnitude than the periplasm, its rate constant is still higher than that of membrane diffusion, indicating its utility in mitigating the cytosolic accumulation of the molecule.

### Efflux reduces intracellular accumulation to below 1 µM in the periplasm and 0.1 µM in the cytosol

**Fig 7:**
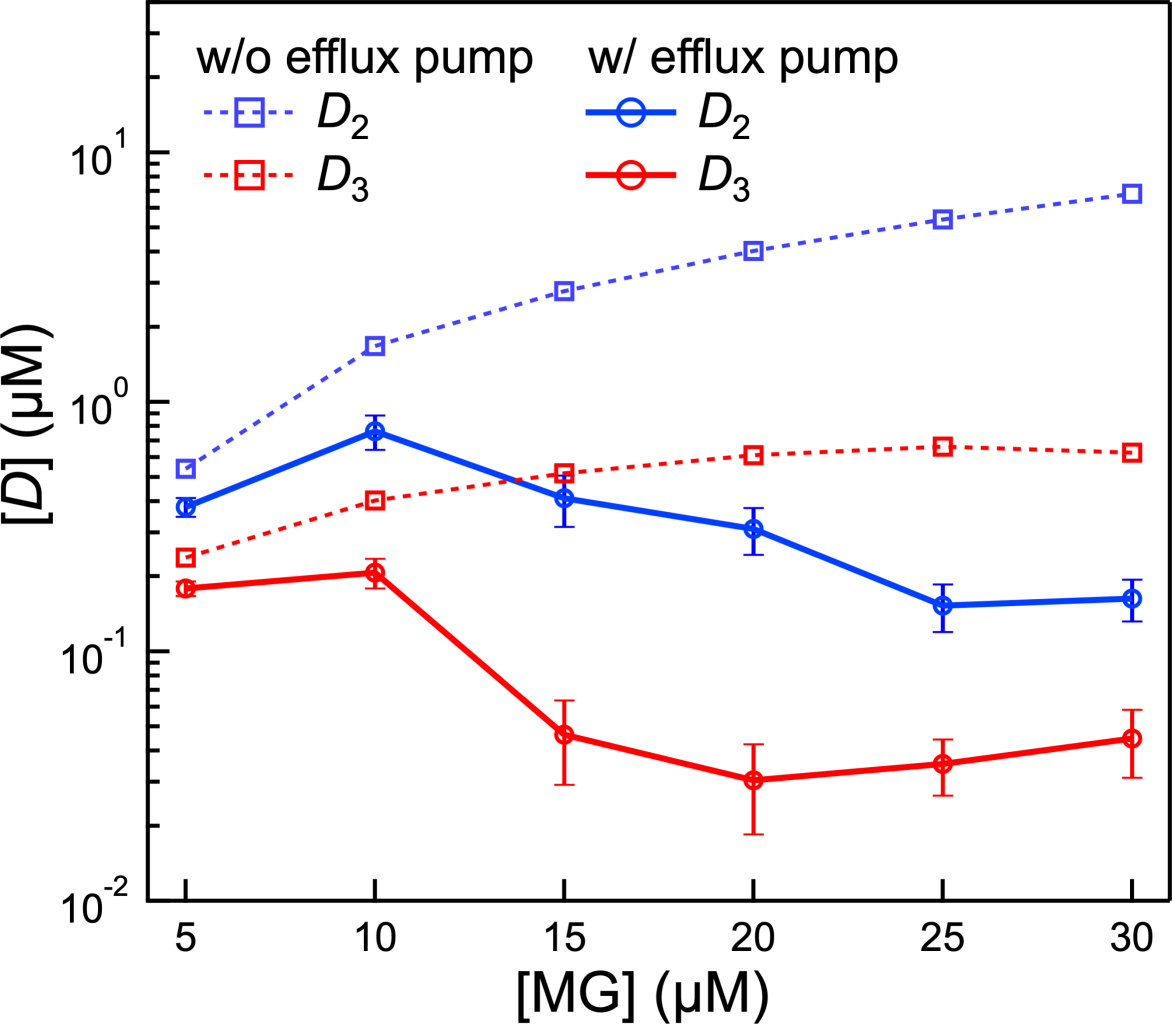
Intracellular concentrations on both sides of the CM for increasing MG concentrations. Blue points are MG concentrations in the periplasm region and red points the cytosol. The points connected by solid lines are concentrations measured with active efflux pumps, while points connected by dashed lines are calculated with the assumption that efflux is not occurring. Error bars for *D*_2_ and *D*_3_ variations are indirectly calculated by adjusting the efflux parameters within their standard deviation range. For the scenario without active efflux pumps, *D*_2_ and *D*_3_ are derived entirely from simulations, so no error bars are shown.

Intracellular accumulation of undesirable substances across the various intracellular domains at high exogenous concentrations illustrates whether the efflux pump is effective. While measuring these concentrations in the periplasm and cytosol is experimentally challenging, our kinetic model allows their determination from fit analysis of the SHS kinetic traces. Figure 7 illustrates the deduced steady-state intracellular concentrations of MG in the periplasmic (*D*_2_) and cytosolic (*D*_3_) entry of the efflux pump. These simulations compare scenarios where the efflux pump is active vs where it is absent. In the latter case, the absence of the efflux pump is simulated by setting both efflux rate constants to zero while keeping all other parameters constant. When the efflux pump is active, concentrations *D*_2_ and *D*_3_ decrease even when the extracellular MG level is high. The comparisons show that the cytosolic efflux pathway reduces the cytosolic concentrations by 20-fold, while the periplasmic efflux pathway is more effective, reducing concentrations in the periplasm by 40-fold. Both pathways effectively reduce intracellular concentrations. The efflux pumps appear to be activated when the MG concentration in the periplasm reaches 1 µM and remain active in order to maintain MG concentrations below this level. For the cytosol, efflux is activated when the MG concentration reaches 0.1 µM (and then maintains the MG concentration below this level). Notably, the pump becomes saturated at high concentrations. For instance, above 25 µM, the periplasmic efflux capacity becomes saturated, leading to an increase in periplasmic concentration. Similarly, when the extracellular concentration exceeds 15 µM, cytosolic efflux capacity is saturated, causing an increase in the cytosolic concentration.

### Efflux simultaneously acts on multiple substrates

The experiments with n-hexane (Fig. 3) show that *P. aeruginosa*’s efflux pump’s capacity for the expulsion of MG is significantly reduced at high hexane concentrations. These findings support the notion that the efflux pump can handle a variety of undesirable molecules, indicating a broad spectrum of action (101). Specifically, the presence of hexane was shown to interfere with the pump’s ability to eject MG, leading to an increased accumulation of MG within the bacterial cells. This suggests that in environments with high concentrations of linear alkanes (such as fuel storage facilities), the efflux pump’s capacity can be overwhelmed, impairing its ability to remove antimicrobials effectively. These observations highlight the pump’s dual role in managing both MG and hexane extrusion but also reveal its limitations in situations where different types of molecules compete to be effluxed. This strategy of reducing bacteria’s defense against the uptake of antimicrobials may be useful for suppressing bacteria growth in fuel storage facilities and prevent the biodegradation of fuels. This competition could also be the explanation for cases where the treatment of n-butanol has been found to facilitate the antibiotic effect of the aminoglycosides (102). Furthermore, these results start to elucidate a potential mechanism by which high-affinity small molecules known as EPIs when added to fuel have been reported to prevent the growth of bacteria and biodeterioration of hydrocarbon fuels (21, 22, 95, 103, 104). It is believed that EPIs may act either by directly plugging the outer membrane component of the RND efflux pump or by entering the cell (i.e., through either the periplasm or cytosol) and competitively inhibiting the extrusion of hydrocarbons, which then accumulate in the cell to toxic levels preventing bacterial growth and eventually killing the cells. The results presented here support the competitive inhibition of hydrocarbon efflux by EPIs as the likely mechanism of action of EPIs in fuel.

### Conclusions

In this study, we used the nonlinear optical technique, second harmonic light scattering, to quantitatively measure the rate constants of efflux of the mild antimicrobial, MG, from the periplasm and the cytosol in *P. aeruginosa*. We observed that the efflux pump plays a significant role in reducing MG’s uptake across the bacterial membranes as the exogenous MG concentration increases. We found that MG is expelled from both the periplasmic and cytosol regions of the cell. The efflux pump is activated to keep the MG concentration in the periplasm at <1 µM and the cytosol <0.1 µM. The primary efflux activity occurs in the periplasmic region, where the efflux rate constant is two orders of magnitude higher than in the cytosol. The quantitative characterization shows that efflux from the periplasm is the first line of defense to eject undesirable molecules. The periplasm efflux rate constant *k*_effluxP_ increases with higher MG concentrations but saturates at concentrations above 25 µM. Similarly, the cytosol efflux rate constant *k*_effluxC_ displays an increasing trend with MG concentration and saturates for concentrations over 15 µM. Our work indicates that RND efflux pumps act as a primary periplasmic protection mechanism against toxic compounds.

High concentrations of n-hexane compete with MG to be effluxed and reduce the rate of ejection of MG from the bacterial cell. This observation suggests a strategy for eradicating bacteria in fuel-rich environments: Uptake of antimicrobials is enhanced even at low antimicrobial concentrations as the efflux pumps are preoccupied with expelling undesirable hydrocarbons. Furthermore, the observation that MG extrusion is mainly affected in the periplasm (which potentially prevents the internalization of MG to the cytoplasm) provides important insight into the uptake, translocation, and localization of EPIs and the mechanisms by which EPIs inhibit the expulsion of hydrocarbons by efflux pumps leading to a biocidal effect. Further research using the SHS approach may lead to the elucidation of the mechanisms of action of EPIs on efflux pumps and their biocidal action in fuels.

## MATERIALS AND METHODS

### Bacteria and solution preparation

Discrete colonies of *P. aeruginosa* (ATCC 39327) are grown on Luria broth agar (Lennox, Sigma-Aldrich) plates. Experimental samples, prepared from single colonies, are cultured (37°C, 150 rpm shaking, ca. 14 h) in 100 mL of Luria–Bertani (LB) broth (Miller, Sigma-Aldrich) to late-log/early stationary phase. Samples are lightly pelletized by centrifugation (1,500 × *g*) for 10 min and washed in 1× PBS (137 mM NaCl, 2.7 mM KCl, 1.46 mM KH_2_PO_4_, and 8.54 mM Na_2_HPO_4_) three times to remove residual LB broth. Washed pellets are resuspended in 1× PBS to a stock cell density with an optical density at 600 nm (OD_600_) of 0.7.

In this study, a concentrated stock solution of MG is prepared by dissolving its oxalate salt (obtained from Sigma-Aldrich) in Milli-Q water. The p*K*_a_ value of MG is 6.9 (92). When its oxalate salt is introduced to water, the solution has a pH of 5.30 ± 0.20, with over 98% of the species present as cationic dye. In the SHS experiment, 1 mL of the cell solution in 1× PBS buffer (pH 7.40 ± 0.15) is mixed with 9 mL of MG aqueous solution. The final solution is weakly alkaline. The hydrolysis of the MG cation into the neutral MG carbinol in a weakly alkaline solution is a slow process. Therefore, during the SHS experiment, MG remained predominantly cationically charged, ensuring that the concentration of MG was equivalent to the concentration of MG cations. Additional details on the hydrolysis of MG in weak alkaline solutions are provided in the supplementary information.

A 1× PBS solution containing 110 µM hexane is prepared by dissolving 3.5 µL of hexane (Sigma-Aldrich) into 250 mL of 1× PBS solution. In the experiment investigating the influence of hexane on MG cation transport, bacterial pellets are resuspended in 1× PBS solution containing hexane. To keep a constant concentration of hexane inside and outside of the cell during MG transport experiments, the MG aqueous solutions used in the SHS experiments also contain hexane at 110 µM concentration.

### Second harmonic light scattering

The SHS experiments utilize the 820-nm fundamental light output from a Ti:sapphire laser (Coherent, Micra V, oscillator only, 76 MHz repetition rate, 96 fs pulse duration, 400 mW average power). This short pulse laser ensures that the pulse energy (5.3 nJ) does not damage the cell sample while the peak pulse power at 55 kW is sufficiently high to facilitate SHG. The laser beam traverses a long pass filter to remove any 410 nm light produced by previous optical surfaces before finally reaching the sample. The laser beam intersects with a liquid jet produced by a liquid flow system, pumping the sample colloidal solution through a circular stainless-steel nozzle with a 1/16″ inner diameter. The laser beam is focused into a 50 µm beam waist inside the colloidal sample, with the laser’s peak pulse intensity density at this cross-section estimated to be 2.79 × 10^9^ W/cm². The focal volume of the laser interacting with the sample is 3 nL. Using a liquid jet as the sample minimized SH signals that might be generated from surfaces or interfaces and the heat effect. The colloidal solution, containing cells, PBS, and chemicals, is continuously circulated through Nalgene tubing (Nalge Nunc, Inc.) from a 10 mL sample reservoir to the inlet of a motorized pump (Micropump, Inc.) and back into the reservoir. The solution is continuously stirred using a magnetic stirrer (Spectrocell, Inc.) to maintain homogeneity. The SH signal is recorded at the forward scattering angle to selectively collect the SH signal. Both fundamental light (820 nm) and SH light (410 nm) scattered from the sample are passed through a BG39 band-pass filter and a monochromator (1 mm entrance and exit slits, 410 ± 1 nm bandwidth). The signal is detected by a photomultiplier (Hamamatsu, R4220) and preamplifier (Stanford Research Systems, SR 440) and processed through a correlated photon counting system (Stanford Research Systems, SRS SR400) with a 0.5 s time resolution.

All time-resolved SHS experiments begin by first measuring the background signal from the hyper-Rayleigh scattering of a 9-mL MG solution without any cells. Once the background is obtained, 1 mL bacteria suspension is added to the flow-jet reservoir at the time denoted as *t* = 0 s. The working cell density of *P. aeruginosa* in the reservoir is 5 × 10^7^ mL^−1^ (OD_600_ ∼ 0.07). Each of the SHS experiments is repeated at least three times, and the average signal and the corresponding uncertainty for each data point are reported. It is important to highlight that the MG cation has an electronic transition near 400 nm that facilitates the resonant enhancement of SHG. In contrast, all other molecules utilized in the experiment (e.g., hexane and PBS buffer) are SHS-inactive and do not generate detectable SHS signals in our experiments.

## Data Availability

All data obtained and analyzed in this study are included in this article and the associated supplemental material.

## SUPPLEMENTAL MATERIAL

Supplemental material aem.01447-24-s0001.docx Supplemental methods, Fig. S1 to S3, and Tables S1 and S2.

ASM does not own the copyrights to Supplemental Material that may be linked to, or accessed through, an article. The authors have granted ASM a non-exclusive, world-wide license to publish the Supplemental Material files. Please contact the corresponding author directly for reuse.
