# Supplementary material for: Quantifying bacterial efflux within subcellular domains of Pseudomonas aeruginosa
Source: Appl Environ Microbiol. 2024 Oct 30;90(11):e01447-24. doi: 10.1128/aem.01447-24 (PMC11577755; doi:10.1128/aem.01447-24)
Supplement: Supplemental material — Supplemental methods, Fig. S1 to S3, and Tables S1 and S2. [file aem.01447-24-s0001.docx]

**Supporting Information- Quantifying Bacterial Efflux within Sub-Cellular Domains of *Pseudomonas aeruginosa***

Yujie Li^1^, Michael J. Wilhelm^1†*^, Tong Wu^1^, Xiao-Hua Hu^1^, Oscar N. Ruiz,^2^ and Hai-Lung Dai^1*^

^1^Department of Chemistry, Temple University, 1901 North 13^th^ street, Philadelphia, PA, USA

^2^Biomaterials Branch, Materials & Manufacturing Directorate, Air Force Research Laboratory, Wright-Patterson AFB, OH 45433-7718, USA

^†^Present address: Chemical Physics and Analysis, Pacific Northwest National Laboratory, Richland, WA 99352, USA

^*^**Corresponding Author**

^*^**Corresponding Author**

Michael J. Wilhelm, E-mail: [michael.wilhelm@alumni.upenn.edu](mailto:michael.wilhelm@alumni.upenn.edu); Hai-Lung Dai, E-mail: [hldai@temple.edu](mailto:hldai@temple.edu)

Table of content:

[1. The equilibrium of the malachite green in alkaline buffer 2](#_Toc160540958)

[2. Kinetic model description 4](#_Toc160540959)

3. Additional data on efflux of hexane……………………………………………………………..7

[**Table S1**. pH of MG Aqueous Solutions in 0.1×PBS Buffer (pH = 7.4) at various concentrations 10](#_Toc160540676)

[**Table S2**. Adsorption densities, adsorption free energies, and transport rate constants deduced as fitting parameters in the complete kinetic model including efflux. 11](#_Toc160540677)

[**Figure S1**. Spectral properties of malachite green oxalate in water at different concentrations. 8](#_Toc176783619)

[**Figure S2.** Spectra and coloration of malachite green oxalate in PBS buffer at diverse pH levels 10](#_Toc176783620)

[**Figure S3.** Measured time-resolved SHS traces for 10 µM and 25 µM MG. 11](#_Toc176783621)

1. The equilibrium of the malachite green in alkaline buffer

Malachite green (MG), possessing a pK_a_ of 6.9(1–3), forms a solution with a pH of 5.30 upon dissolution of its oxalate salt in water, with the predominant species being cationic, accounting for over 98% of the population. With an increase in the concentration of the MG solution, the proportion of cationic species rises, resulting in a deeper blue color. This correlation is corroborated by **Figure S1**. Additionally, spectral analysis of MG at varying concentrations reveals no formation of dimers, even as the concentration escalates.

As the pH of the solution increases and hydroxyl ions become prevalent, a hydrolysis reaction ensues between the blue cationic species and the colorless carbinol species, leading to a progressive decolorization. The detailed mechanistic elucidation can be referred to S1 and S2, which illustrate the nucleophilic attack of a hydroxyl ion on the electrophilic carbon of malachite green, thereby disrupting its conjugated system and culminating in the formation of a colorless carbinol base species(4, 5). The MG carbinol base (MGCB) spectrum has much lower absorption coefficients at 415 nm and 600 nm compared with MG at the same concentration, supporting the idea that the MGCB is colorless (6). It’s worthwhile to note that the reaction rate is linearly proportional to the hydroxyl ion concentration, when the hydroxyl ion concentration is low, it takes several hours until the equilibrium is reached (1, 3).

The coloration of malachite green (MG) solutions serves as an effective metric for quantifying the concentration of cations present. To corroborate the hypothesis that MG decolorizes slowly in weakly alkaline buffers due to hydrolysis, a spectral study is conducted. MG solutions of identical concentration are prepared in buffers with pH values of 6, 7, and 8. According to MG's pKa and the calculations presented in equation S3, it is anticipated that the solutions at pH 6, 7, and 8 would contain approximately 90%, 50%, and 10% cationic species, respectively. Initially, all MG samples exhibit uniform color and similar UV-Vis absorbance profiles, as depicted in **Figure S2** (a), a finding that seems incongruent with the expected cation percentages. However, after around four hours, significant fading is observed in MG solutions at mildly alkaline pH while in acidic buffer, there is only minimal fading. This coloration difference is highlighted in **Figure S2** (b). The MG solutions in weak alkaline conditions show a notable decrease in absorbance at 415 nm and 600 nm, as detailed in **Figure S2**(b).

The pH of MG solutions in 0.1×PBS Buffer (pH = 7.4) at various concentrations is recorded in **Table S1**, which further support that the PBS buffer provides mild alkaline environment, and the pH condition is proper for the bacterial keeping healthy morphology during the experiment. Further, **Figure S2** (c) tracks the color and pH changes over time in a 9:1 mixture of MG solution and 1 × PBS buffer at pH 7.4. Immediately after mixing, the pH of MG solution shifts from 5.3 to 7, creating a mildly alkaline environment. For the first 50 minutes, there is no change in color; from 50 to 150 minutes, a slight fading is observed, and after 3 to 4 hours, the color significantly fades.

In our SHS experiment, which investigates MG uptake across cell membranes and lasts only 20 minutes in mildly alkaline conditions, the above findings suggest the presence of predominantly MG cations in the MG solution. Hence, we can infer that the concentration of cations matches the dye concentration in the solution.

(S2)

$pKa-pH=log\left( \frac{\left[ {MG}^{+} \right]}{\left[ MG\cdot OH \right]} \right)\ldots\ldots\ldots\ldots\ldots\ldots\ldots\ldots\ldots\ldots\ldots\ldots\ldots.$ (S3)

1. Kinetic model description

Our kinetic model describes the interaction between the molecules and the cell membrane when molecules are adsorbed on the surface and transported across the membrane (7–9). *D*_i_ define the concentration of MG in region i (*D*_0_ is the external MG concentration; *D*_1_ is the MG concentration in the region between the outer membrane and peptidoglycan mesh; *D*_2_ is the MG concentration in the region between the peptidoglycan mesh and inner membrane; *D*_3_ is the MG concentration in the region of cytosol). *N*_j,k_ is the surface concentration of the k*^th^* surface of the j^th^ membrane (i.e., *N*_1,o_ refers to the outer leaflet of the OM, *N*_1,i_ is the inner leaflet of the OM, *N*_2,o_ is the outer leaflet of the CM, and *N*_2,i_ is the inner leaflet of the CM); and *E*_j.k_ denotes empty surface sites, defined by the maximum number of surface sites *(*$N_{j,k}^{max}$ ) less the occupied sites (i.e., *E*_j,k_ *=* $N_{j,k}^{max}$ *– N*_j,k_). Actually, prior to MG occupation, surface sites are never empty, but rather saturated with water. Subsequently, the adsorption of MG onto a membrane surface site results in the release of a bound water molecule. Using these definitions, a series of kinetic equations describing molecular uptake can be generated by considering of the following steps:

1. Adsorption and desorption from the outer leaflet of OM, where *k*_a1_ and *k*_d1_ are rate constants for MG cation adsorbing or desorbing on the outer leaflet of outer membrane, respectively.

1. Transport across the outer membrane protein (OMP), where *k*_OMP_ is the rate constant for crossing outer membrane facilitated by the porin channels. The left-right arrow indicates that the reaction is reversible.

$D_{0}{\underset{\leftrightarrow}{k_{\mathrm{OMP}}} D}_{1}\ldots\ldots\ldots\ldots\ldots\ldots\ldots\ldots\ldots\ldots\ldots\ldots\ldots\ldots\ldots.\ldots.(S5)$

1. Direct transport through the outer lipid membrane, where *k*_mem_ is the rate constant for crossing the outer membrane through passive diffusion.

$$N_{1,o}+E_{1,i}\underset{\leftrightarrow}{k_{mem}}N_{1,i}+E_{1,o}\ldots\ldots\ldots\ldots\ldots\ldots\ldots\ldots\ldots\ldots\ldots..(S6)$$

2. Adsorption and desorption from the inner leaflet of the OM, where *k*_a2_ and *k*_d2_ are rate constants for MG cation adsorbing or desorbing on the inner leaflet of outer membrane, respectively.

$D_{1}+E_{1,i}\begin{matrix} k_{a2} \\ \rightleftarrows\\ k_{d2} \end{matrix}N_{1,i}+H_{2}O \ldots\ldots\ldots\ldots\ldots\ldots\ldots\ldots\ldots\ldots\ldots\ldots\ldots..(S7)$

1. Diffuse through the PM, where *k*_PM_ is the rate constant for MG cation diffusion across the peptidoglycan mesh.

$$D_{1}\underset{\leftrightarrow}{k_{\mathrm{PM}}}D_{2} \ldots\ldots\ldots\ldots\ldots\ldots\ldots\ldots\ldots\ldots\ldots\ldots\ldots\ldots\ldots\ldots\ldots. (S8)$$

2. Adsorption and desorption from the outer leaflet of the inner membrane, where *k*_a3_ and *k*_d3_ are rate constants for MG cation adsorbing onto or desorbing from the outer leaflet of inner membrane, respectively.

$$D_{2}+E_{2,o}\begin{matrix} k_{a3} \\ \rightleftarrows\\ k_{d3} \end{matrix}N_{2,o}+H_{2}O \ldots\ldots\ldots\ldots\ldots\ldots\ldots\ldots\ldots\ldots\ldots\ldots\ldots(S9)$$

3. Direct transport through the inner membrane, where *k*_mem_ is the rate constant for crossing the inner membrane lipid bilayer through passive diffusion.

$$N_{2,o}+E_{2,i}\underset{\leftrightarrow}{k_{mem}}N_{2,i}+E_{2,o}\ldots..\ldots\ldots\ldots\ldots\ldots\ldots\ldots\ldots\ldots(S10)$$

4. Adsorption and desorption from the inner leaflet of the inner membrane, where *k*_a4_ and *k*_d4_ are rate constants for MG cation adsorbing or desorbing on the inner leaflet of inner membrane, respectively.

$$D_{3}+E_{2,i}\begin{matrix} k_{a4} \\ \rightleftarrows\\ k_{d4} \end{matrix}N_{2,i}+H_{2}O \ldots\ldots\ldots\ldots\ldots\ldots\ldots\ldots\ldots\ldots\ldots\ldots\ldots(S11)$$

5. Additional to that, the equations below are used to describe the efflux of molecules from either the periplasm or cytosol to externally.

$D_{2}\underset{\to}{k_{\mathrm{effluxP}}} D_{0} \ldots\ldots\ldots\ldots\ldots\ldots..\ldots\ldots\ldots\ldots\ldots\ldots\ldots\ldots(S12)$

$$D_{3}\underset{\to}{k_{\mathrm{effluxC}}} D_{0}\ldots\ldots\ldots\ldots\ldots\ldots..\ldots\ldots\ldots\ldots\ldots\ldots\ldots\ldots(S13)$$

Since the uptake of MG across the *P. aeruginosa* is considered as passive diffusion, it can assume that transport across the OMP, the peptidoglycan mesh (PM), and the lipid membranes are completely reversible. In addition, the adsorption and desorption to the inner leaflet of the OM and both leaflets of the CM likely occur with a common rate (i.e., adsorption: *k*_a2_ *= k*_a3_ *= k*_a4_, desorption: *k*_d2_ *= k*_d3_ *= k*_d4_). The adsorption and desorption rates, however, need not be equal (*k*_aj_ *≠ k*_dj_). Applying these simplifying assumptions, the eight equilibriums above can be used to generate a series of coupled differential equations which describe the time-varying evolution of the bulk concentrations and surface concentrations (i.e., *D*_0_*, D*_1_*, D*_2_*, D*_3_*, N*_1,o_*, N*_1,i_*, N*_2,o_*, N*_2,i_):

$$\frac{\partial D_{0}}{\partial t}=-k_{a1}D_{0}\left( N_{1,o}^{max}-N_{1,o} \right)-k_{OMP}\left( D_{0}-D_{1} \right)+k_{d1}N_{1,o}\left[ H_{2}O \right]+k_{\mathrm{effluxP}}\left[ D_{2} \right]+k_{\mathrm{effluxC}}\left[ D_{3} \right]\ldots\ldots\ldots\ldots.. (S14)$$

$$\frac{\partial D_{1}}{\partial t}=-k_{a2}D_{1}\left( N_{1,i}^{max}-N_{1,i} \right)+k_{OMP}\left( D_{0}-D_{1} \right){-k_{PM}\left( D_{1}-D_{2} \right)+k}_{d2}N_{1,i}\left[ H_{2}O \right]\ldots\ldots\ldots\ldots\ldots\ldots\ldots\ldots\ldots\ldots\ldots. .\left( S15 \right)$$

$$\frac{\partial D_{2}}{\partial t}=-k_{a3}D_{2}\left( N_{2,o}^{max}-N_{2,o} \right){+k_{pm}\left( D_{1}-D_{2} \right)+k}_{d3}N_{2,o}\left[ H_{2}O \right]-k_{\mathrm{effluxP}}\left[ D_{2} \right]\ldots\ldots\ldots\ldots\ldots\ldots\ldots\ldots\ldots\ldots\ldots\ldots\ldots\left( S16 \right)$$

$$\frac{\partial D_{3}}{\partial t}=-k_{a4}D_{3}\left( N_{2,i}^{max}-N_{2,i} \right){+k}_{d4}N_{2.i}\left[ H_{2}O \right]-k_{\mathrm{effluxC}}\left[ D_{S} \right]\ldots\ldots\ldots\ldots\ldots\ldots\ldots\ldots\ldots..\ldots\ldots\ldots\ldots\ldots\ldots\ldots\ldots\ldots\ldots\ldots\left( S17 \right)$$

$$\frac{\partial N_{1,o}}{\partial t}=k_{a1}D_{0}\left( N_{1,o}^{max}-N_{1,o} \right)-k_{mem}\left( N_{1,o}-N_{1,i} \right)-k_{d1}N_{1,o}\left[ H_{2}O \right]\ldots\ldots\ldots\ldots\ldots\ldots\ldots\ldots\ldots\ldots\ldots\ldots\ldots\ldots\ldots\ldots\ldots.\left( S18 \right)$$

$$\frac{\partial N_{1,i}}{\partial t}=k_{a2}D_{1}\left( N_{1,i}^{max}-N_{1,i} \right)+k_{mem}\left( N_{1,o}-N_{1,i} \right)-k_{d2}N_{1,i}\left[ H_{2}O \right]\ldots\ldots\ldots\ldots\ldots\ldots\ldots\ldots\ldots\ldots\ldots\ldots\ldots\ldots\ldots\ldots\ldots\ldots(S19)$$

$$\frac{\partial N_{2,o}}{\partial t}=k_{a3}D_{2}\left( N_{2,o}^{max}-N_{2,o} \right)-k_{mem}\left( N_{2,o}-N_{2,i} \right)-k_{d3}N_{2,o}\left[ H_{2}O \right]\ldots\ldots\ldots\ldots\ldots\ldots\ldots\ldots\ldots\ldots\ldots\ldots\ldots\ldots\ldots\ldots.. .\left( S20 \right)$$

$$\frac{\partial N_{2,i}}{\partial t}=-k_{a4}D_{3}\left( N_{2,i}^{max}-N_{2,i} \right)+ k_{mem2}\left( N_{2,o}-N_{2,i} \right)-k_{d4}N_{2,i}\left[ H_{2}O \right]\ldots\ldots\ldots\ldots\ldots\ldots\ldots\ldots\ldots\ldots\ldots\ldots. \ldots\ldots\ldots\ldots.\left( S21 \right)$$

The solutions to the ordinary differential equations yield the base components (i.e., *D*_0_*, D*_1_*, D*_2_*, D*_3_*, N*_1,o_*, N*_1,i_*, N*_2,o_*, N*_2,i_) which describe the time-dependent responses observed in the SHS. Specifically, the time resolved SHS response can be described as the square of a combination of the surface concentrations:

1. Additional data on efflux of hexane

Additional MG / hexane experiments have been conducted to show the competition on efflux behavior between the MG and hexane. Observations made with MG concentrations of 10 µM and 25 µM (**Figure S3**), show a similar trend where the 110 µM hexane has no effect on MG transport at 10 µM but a substantial effect at 25 µM. This result clearly shows that the hexane at high concentration competes against MG at high concentration for efflux actions.

### Figures

**Figure S1**. Spectral properties of malachite green oxalate in water at different concentrations, captured using a Jasco V 630 UV-Vis/NIR spectrophotometer in Eppendorf™ Vis Cuvettes with a 1-cm path length. The bandwidth for the spectra is set at 1 nm. The absorbance peaks at 410 nm and 615 nm, associated with the cationic species, rise with increasing concentration. It shows the UV-vis spectral changes associated with these concentrations. The spectra highlight the increase at the 615 nm and 410 nm peaks from the MG cation as the concentration increases. The linear increase indicates the absence of any byproduct of MG compound, such as MG dimer.

**
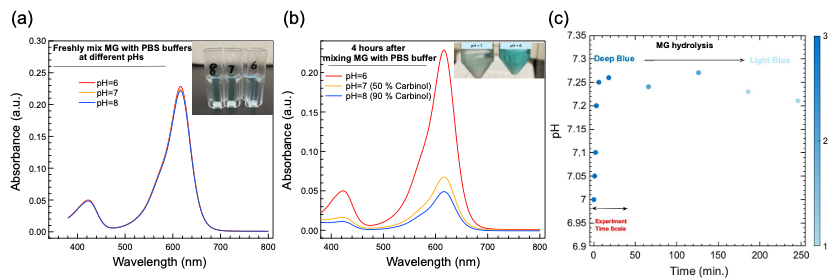
**

**Figure S2.** Spectra and coloration of malachite green oxalate in PBS buffer at diverse pH levels under the following conditions: (a) immediately after preparation of the solutions; (b) 4 hours after the preparation; (c) observing the changes in color and pH over time for a mixture composed of 9 mL of MG solution and 1 mL of PBS buffer at pH 7.4. The color bar (1-3) indicates the darkness of blue, with 1 representing the lightest blue and 3 representing the deepest blue.


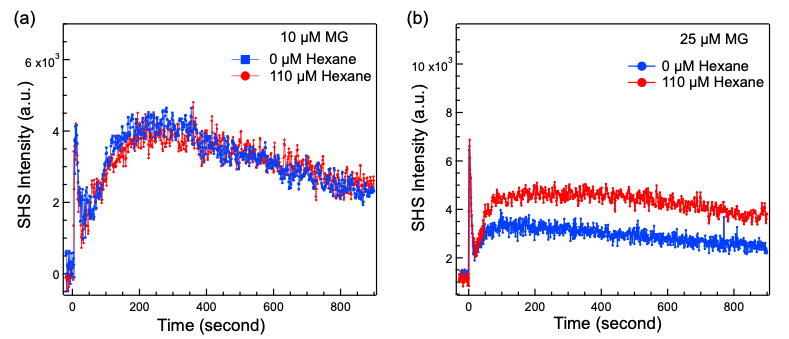


**Figure S3.** Measured time-resolved SHS traces for (a) 10 µM (3.3 µg/mL), and (b) 25 µM (8.2 µg/mL) MG. In the panel, red points represent cells pretreated with 110 µM hexane, whereas blue points are from cells untreated with hexane.

### Tables

**Table S1**. pH of MG aqueous solutions in 0.1×PBS Buffer (pH = 7.4) at various concentrations

| MG Concentration(µM) | 5 | 10 | 15 | 20 | 25 | 30 | 1×PBS |
| --- | --- | --- | --- | --- | --- | --- | --- |
| pH | 7.35 | 7.33 | 7.28 | 7.27 | 7.17 | 7.12 | 7.37 |

**Table S2**. Adsorption densities, adsorption free energies, and transport rate constants deduced as fitting parameters in the complete kinetic model including efflux, for the data in **Figure 1**. The error bar corresponds to the standard deviation obtained from the fitting

| [MG^+^] (µM) | 5 | 10 | 15 | 20 | 25 | 30 |
| --- | --- | --- | --- | --- | --- | --- |
| $N_{1,o}^{Max}$ (µM)^a,b,c^ | 4.40 ± 0.09 | 4.40 ± 0.09 | 4.40 ± 0.09 | 4.40 ± 0.09 | 4.40 ± 0.09 | 4.40 ± 0.09 |
| $N_{1,i}^{Max}$ (µM)^a,h,c^ | 3.85± 1.08 | 3.85± 1.08 | 3.85± 1.08 | 3.85± 1.08 | 3.85± 1.08 | 3.85± 1.08 |
| $N_{2,o}^{Max}$ (µM)^a,b,c^ | 2.84 ± 1.12 | 2.84 ± 1.12 | 2.84 ± 1.12 | 2.84 ± 1.12 | 2.84 ± 1.12 | 2.84 ± 1.12 |
| $N_{2,i}^{Max}$ (µM)^a,b,c^ | 2.61 ± 1.12 | 2.61 ± 1.12 | 2.61 ± 1.12 | 2.61 ± 1.12 | 2.61 ± 1.12 | 2.61 ± 1.12 |
| ∆G_1,o_ (kcal/mol)^a,b,c^ | -9.56± 0.08 | -9.56± 0.08 | -9.56± 0.08 | -9.56± 0.08 | -9.56± 0.08 | -9.56± 0.08 |
| ∆G_1,i_ (kcal/mol)^a,b,c^ | -9.82± 0.10 | -9.82± 0.10 | -9.82± 0.10 | -9.82± 0.10 | -9.82± 0.10 | -9.82± 0.10 |
| ∆G_2_ (kcal/mol)^a,b,c^ | -10.40±0.54 | -10.40±0.54 | -10.40± 0.54 | -10.40± 0.54 | -10.40 ± 0.54 | -10.40±0.54 |
| *k*_OMP_ (×10^−2^ s^–1^)^a,b,c^ | 7.90 ± 0.35 | 7.90 ± 0.35 | 7.90 ± 0.35 | 7.90 ± 0.35 | 7.90 ± 0.35 | 7.90 ± 0.35 |
| *k*_mem_ (× 10^−3^ s^−1^)^a,b,c^ | 1.10 ± 0.46 | 1.10 ± 0.46 | 1.10 ± 0.46 | 1.10 ± 0.46 | 1.10 ± 0.46 | 1.10 ± 0.46 |
| *k*_PM_ (× 10^−2^ s^−1^)^a,b,c^ | 2.49 ± 0.27 | 2.49 ± 0.27 | 2.49 ± 0.27 | 2.49 ± 0.27 | 2.49 ± 0.27 | 2.49 ± 0.27 |
| *k*_effluxP_ (s^−1^) | (3.54±0.70)×10^−2^ | (4.66±1.36)× 10^−2^ | 0.20±0.05 | 0.45±0.10 | 1.26±0.26 | 1.23±0.29 |
| *k*_effluxC_ (s^−1^) | (2.25±0.50)× 10^−6^ | (0.99 ± 0.20) × 10^−3^ | (4.68 ±1.01) × 10^−3^ | (4.82±1.11) × 10^−3^ | (4.99±1.24) × 10^−3^ | (5.02 ± 1.08) × 10^−3^ |

Note: a. The $N_{i,j}^{Max}$represent the maximum number density of the adsorption sites at the *i^th^* surface of the j^th^ membrane (i.e., *N*_1,o_ refers to the outer leaflet of the OM, *N*_1,i_ is the inner leaflet of the OM, *N*_2,o_ is the outer leaflet of the CM, and *N*_2,i_ is the inner leaflet of the CM);

b*. ∆G_i,j_* represents the Gibbs free energy for adsorption equilibrium for at the *i^th^* surface of the j^th^ membrane. $\Delta G = -RT\ln K$, where $K=\frac{k_{a}}{k_{d}}$. *k*_a_ and *k*_d_ is the adsorption and desorption rate constant at each leaflet of the membrane, and these processes are depicted in **Figure 2**;

c. The fitting of these traces is conducted by means of global fitting where all the parameters, along with their respective standard deviations, are consistent across the concentrations, except for parameters *k*_effluxP_ and the *k*_effluxC_.

### Reference

1. Alderman DJ. 1985. Malachite green: a review. J Fish Dis 8:289–298.

2. Culp SJ, Beland FA. 1996. Malachite Green: A Toxicological Review. Int J Toxicol 15:219–238.

3. Goldacre RJ, Phillips JN. 1949. The ionization of basic triphenylmethane dyes. Journal of the Chemical Society (Resumed) 7:1724–1732.

4. Felix LD. 2017. Kinetics of the reaction between malachite green and hydroxyl ion in the presence of reducing sugars. Journal of Chemical Technology and Metallurgy 52:526–531.

5. Felix LD, Adesoji A. 2017. Kinetics and thermodynamic study of alkaline fading of malachite green in aqueous solution. Journal of Applied and Fundamental Sciences 3:52.

6. Li G, Magana D, Dyer RB. 2012. Direct observation and control of ultrafast photoinduced twisted intramolecular charge transfer (TICT) in triphenyl-methane dyes. Journal of Physical Chemistry B 116:12590–12596.

7. Zeng J, Eckenrode HM, Dounce SM, Dai HL. 2013. Time-resolved molecular transport across living cell membranes. Biophys J 104:139–145.

8. Wilhelm MJ, Sheffield JB, Gonella G, Wu Y, Spahr C, Zeng J, Xu B, Dai HL. 2014. Real-time molecular uptake and membrane-specific transport in living cells by optical microscopy and nonlinear light scattering. Chem Phys Lett 605–606:158–163.

9. Wu T, Wilhelm MJ, Li Y, Ma J, Dai HL. 2022. Indole Facilitates Antimicrobial Uptake in Bacteria. ACS Infect Dis 8:1124–1133.
